# Partitioning additive genetic variance into genomic and remaining polygenic components for complex traits in dairy cattle

**DOI:** 10.1186/1471-2156-13-44

**Published:** 2012-06-13

**Authors:** Just Jensen, Guosheng Su, Per Madsen

**Affiliations:** 1 Department of Molecular Biology and Genetics, Centre for Quantitative Genetics and Genomics, Aarhus University, Research Centre Foulum, DK-8830, Tjele, Denmark

**Keywords:** Genomic variance, Polygenic variance, Chromosomes, Complex traits, Dairy cattle genetic markers

## Abstract

**Background:**

Low cost genotyping of individuals using high density genomic markers were recently introduced as genomic selection in genetic improvement programs in dairy cattle. Most implementations of genomic selection only use marker information, in the models used for prediction of genetic merit. However, in other species it has been shown that only a fraction of the total genetic variance can be explained by markers. Using 5217 bulls in the Nordic Holstein population that were genotyped and had genetic evaluations based on progeny, we partitioned the total additive genetic variance into a genomic component explained by markers and a remaining component explained by familial relationships. The traits analyzed were production and fitness related traits in dairy cattle. Furthermore, we estimated the genomic variance that can be attributed to individual chromosomes and we illustrate methods that can predict the amount of additive genetic variance that can be explained by sets of markers with different density.

**Results:**

The amount of additive genetic variance that can be explained by markers was estimated by an analysis of the matrix of genomic relationships. For the traits in the analysis, most of the additive genetic variance can be explained by 44 K informative SNP markers. The same amount of variance can be attributed to individual chromosomes but surprisingly the relation between chromosomal variance and chromosome length was weak. In models including both genomic (marker) and familial (pedigree) effects most (on average 77.2%) of total additive genetic variance was explained by genomic effects while the remaining was explained by familial relationships.

**Conclusions:**

Most of the additive genetic variance for the traits in the Nordic Holstein population can be explained using 44 K informative SNP markers. By analyzing the genomic relationship matrix it is possible to predict the amount of additive genetic variance that can be explained by a reduced (or increased) set of markers. For the population analyzed the improvement of genomic prediction by increasing marker density beyond 44 K is limited.

## Background

Low cost genotyping of individuals or families using genomic markers with constantly increasing density is currently being introduced in genetic improvement programs for agricultural animal and crop species. Use of dense genomic markers can increase the accuracy of predicting additive genetic merit especially for selection of candidates that do not yet have own or progeny records [1]. Such application of dense genomic markers, usually called genomic selection (GS), can reduce the cost of running an intensive breeding program due to potential reductions in the number of individuals tested for own or progeny performance, a shorter generation interval [2], and simultaneously greatly enhance the genetic gain from the program without a concomitant extra increase in the accumulation of inbreeding [3]. Currently, this has led to implementation of GS methods in many large scale commercial dairy cattle breeding programs [4-6]. Implementation in other animal and plant species are being initiated and is expected to increase considerably in the near future [7].

The current industry standard in dairy cattle breeding is use of 50 K chips such as the Illumina Bovine SNP50 BeadChip [8] but other options, ranging from low density 3 K chips to high density 800 K chips, are also commercially available. Increasing the density of genetic markers is expected to increase the amount of genetic variance that can be explained by markers due to increased linkage disequilibrium between markers and causative loci [9]. A further step is the use of complete sequencing of individual genomes either based on direct sequence data or based on sequence data for selected individuals and imputing genotypes for animals that has been genotyped with lower marker density

In human genetics very high density chips have been used in large scale studies [10]. However, genome wide association studies (GWAS) have generally not been able to identify individual genes that can explain a large proportion of total genetic variance of complex traits recorded in humans. A typical model trait in humans is height that has been investigated in many studies. This is a trait measured with an accuracy that is comparable to the accuracy of daughter yield deviations of dairy sires that are progeny-tested using large daughter groups. Height in humans is known to have a heritability around 0.8 [11]. In several well designed large scale studies with the use of very high density SNPs, numerous loci have been identified that are significantly associated with human height, yet each of them typically only account for a very small fraction of total phenotypic variance. Collectively these loci are only responsible for up to 5% of the total phenotypic variance in human height [11,12]. This has lead to discussions among human geneticists about the missing heritability of complex traits [13]. Suggested explanations have included dominance and epistatic interactions, genotype by environment interactions and common epigenetic factors causing resemblance between relatives [14].

As mentioned above the total variance explained by previously identified causal loci is usually only a small fraction of total genetic variance in the populations investigated. In GWAS very stringent significance thresholds are necessary due to the very large number of statistical tests that are conducted when searching the whole genome using high density SNP marker panels. This will only allow loci with large effects to become statistically significant. However, [10] showed that a considerable proportion of additive genetic variance can be explained by a very large number of SNPs and their effects can be predicted simultaneously using appropriate statistical models. Such models also includes the SNPs with small effects as long as they are associated with the trait of interest This association may be either due to SNPs being located within causative loci, being in linkage disequilibrium (LD) with causative genes, or due to markers tracing parts of familial additive genetic relationships among genotyped animals [15]. These results are well in line with results from dairy cattle where a large proportion of additive genetic can be explained using dense markers.

If not all genetic variance can be explained by markers then, in order to ensure optimal predictions, the remaining genetic variance should be accounted for in other ways. A simple approach is to combine predicted breeding values based on genomic information with traditional breeding values based on pedigree using selection index theory [16]. An alternative method is to include both genomic and pedigree relationships in the analysis simultaneously. Previous studies have report that a model including a residual polygenic effect slightly increases reliability and reduce bias in prediction of future records [17,18]. Such a method requires the partitioning of genetic variance accounted for by genomic information and remaining genetic variance accounted for by pedigree relationships.

Recently, [19,20], proposed a one-step method combining marker based genomic relationships and pedigree-based relationships into a single relationship matrix. An important factor in this procedure is that marker-based and pedigree-based relationship coefficients must be expressed on the same scale, i.e. the variance of the genomic and the classical additive genetic effects must refer to the same base population, and the proportion of total genetic variance explained by markers and the remaining genetic variance must be known. Currently implemented multistep procedures may also need to be on the same scale in order to ensure derivation of optimum combined predictions.

The purpose of this study was to evaluate the amount of additive genetic variation in production and fitness related traits in dairy cattle, to quantify the amount of additive genetic variation that can be explained using genomic markers with different density, and to quantify the amount of genomic variance that can be ascribed to individual chromosomes. The value of increasing the density of marker information for predicting genetic merit was also assessed using subsets of available markers.

## Methods

### Data

Data on deregressed proofs (**DRP**) were used as response variable in the present study, which were derived from the Nordic Holstein genetic evaluations official spring 2011 run. Production traits included milk production (***milk***), fat production (***fat***), protein production (***protein***). Fitness related traits included female fertility (***fertility***), other diseases than mastitis (***health***), and mastitis (***mastitis***). The specific trait definitions and recording procedures are detailed in http://www.nordicebv.info/Forside.htm and [21]. The Nordic Genetic Evaluation (NAV) system for dairy cattle includes all recorded dairy cattle in Denmark, Sweden and Finland and published proofs is standardized so that the variance of predicted breeding values for bulls belonging to a recent two birth year cohort was 100. Since the reliabilities of published proofs vary among traits the variance of the deregressed proofs also vary among the traits analyzed. The deregression removes the shrinkage of the published proofs and the DRP, therefore, is similar to progeny group means corrected for all non-genetic effects influencing the traits analyzed. The ratios of variance components are dependent of the accuracy of the individual DRP. All estimates of variance ratios etc. are shown at the average reliability of the bulls included in the study.

The deregressed proofs were merged with marker records of individual bulls that were typed using Illumina Bovine SNP50 BeadChip (Illumina, San Diego, California, US). Only bulls with both genotype records and deregressed proof for at least one trait in the database were included in the analysis. A summary of the data used are shown in Table 1. In total there were 5217 bulls which were born in the period from 1989 to 2006.

**Table 1:** Mean and standard deviation of deregressed bull proofs

**Abbreviation**	**Trait**	**n**	**mean**	**Sd**
***Milk***	Milk yield	4398	97.41	13.21
***Fat***	Fat yield	4398	96.99	12.23
***Protein***	Protein yield	4398	95.44	14,55
***Fertility***	Female fertility	4415	99.44	16.90
***Health***	Health index	4240	96.69	19.22
***Mastitis***	Mastitis resistance	4398	95.98	11.70

A total of 47152 SNP markers were available in the raw marker data, and after removing markers with minor allele frequency (MAF) < 0.01 and non-informative markers that were a simple linear function of another marker, a total of 44012 markers remained for analysis. A sire-dam pedigree of all bulls included in the analysis was constructed from the official pedigree file from NAV (http://www.nordicebv.info). The pedigree file included a total of 42144 animals and most bulls were traced to the middle of the previous century or earlier.

### Models

The data were analyzed using the following models:

(1) $$y=1\mu +Z_{a}a_{1}+e_{1}$$

(2) $$y=1\mu +Z_{g}\;g_{2}+e_{2}$$

(3) $$y=1\mu +Z_{g}\;g_{3}+Z_{a}\;a_{3}+e_{3}$$

(4) $$y=1\mu +Z_{g}\;g_{c}+Z_{g}\;g_{o}+Z_{a}\;a_{4}+e_{4}$$

where **μ** is the general mean, **a**_*x*_ is the vectors of additive genetic effects not accounted for by genetic markers (for model 1 this reduces to the classical individual animal model since no markers are included in the model), **g**_*x*_ is the vector of the additive genetic effects accounted for by markers, **g**_*c*_ is the vector of additive genetic effects accounted for by markers on a specific autosomal chromosome and **g**_*o*_ is the vector of additive genetic effects accounted for by markers on all remaining chromosomes. 1 is a vector of ones and **Z**_*a*_ and **Z**_*g*_ are incidence matrices relating observations in **y** to additive genetic effects in **a** and in **g**_*x*_, **g**_*c*_ or **g**_*o*_, respectively. Subscript x on vectors in different models indicates that definitions vary with model. Due to computational constraints specific effects of markers on single chromosomes was estimated for only one chromosome at a time, and each analysis included effects of markers on the specific chromosome and the combined effects of markers on all other chromosomes. Therefore model (4) was run one time for each trait and for each chromosome.

The parameter **μ** was considered as a fixed effect in all models and all other effects were assumed random normally distributed effects with variances$\mathrm{var}(a_{x})=A\sigma_{a}^{2}$, where **A** is the additive genetic relationship matrix computed from the full pedigree, and$\mathrm{var}(g_{x})=G\sigma_{g}^{2}$, where

(5) $$G=\frac{(M-P){\left(M-P\right)}^{'}}{2\sum_{j=1}^{m}p_{j}(1-p_{j})}$$

In (5) M is an allele sharing matrix containing the number of copies of the second allele, and P is a matrix containing twice the population frequency of the second allele, i.e.$2p_{j}$, and m is the number of marker loci included in computation of G_._ The division with $2\sum_{j=1}^{m}p_{j}(1-p_{j})$ in (5) is included to ensure that the scale of **A** and **G** are comparable but will otherwise not influence the predictions from the model but only the scale of model parameters. Finally,$\mathrm{var}(e)=D\sigma_{e}^{2}$ where **D** is a diagonal matrix containing weights proportional to the effective number of records in each **DRP**. The linear models (1)-(4) are based on different ways of describing the relationship among animals. For genomic relationships the methods used are detailed in [16]. In short, the relationship matrix **A**, based on pedigree information uses probability of identity by descent, whereas the genomic relationship matrix **G** based on marker information use probability of identity by state.

The number of markers included in computation of **G** in models (2) and (3) were 44012. The same markers were used in model (4) but markers were split into markers for one chromosome at a time and the markers on all other chromosomes pooled such that two genomic relationship of same size were computed. Model (4) was used in this way for all 29 autosomal chromosomes.

All analysis including estimation of variance components using Restricted Maximum Likelihood were conducted using the DMU software [22,23].

### Analysis of genomic relationships

Additive genetic differences between individuals are due to, generally unknown, causative genes. If the genotypes at all causative loci were known, the true genomic relationship matrix (**G**_*t*_) with regard to the trait of interest could be computed based on all the causative loci. In practice this is not possible and instead we compute **G** based on the marker data only and here we name this **G**_*m*_. The accuracy of **G**_*m*_ to describe the genetic covariance among individuals sharing the same causative genes (**G**_*t*_) depends on the linkage disequilibrium between the markers and the causative genes. The accuracy of the genomic relationship matrix can be assessed using the procedure of [10] who regard **G**_*m*_ as an estimate of **G**_*t*._ The procedure includes the following steps:

1. Randomly sample 2 N SNPs across the genome and divide them in two groups of equal size.

2. Calculate **G**_*m*_ using then SNPs in group one and calculate **G**_*t*_ using the SNPs in group two, assuming that the SNPs in group two are the causal variants.

3. Use linear regression

(6) $$g_{t_{\mathrm{jk}}}=\alpha +\beta g_{m_{\mathrm{jk}}}+e$$

for *j ≤ k*. The diagonal dominance of **G**_*t*_ and **G**_*m*_ is removed by subtracting 1.0 from the diagonal elements before estimating α and β.

This procedure is repeated for different N and the relation between *β* and N can be determined empirically.

To obtain an unbiased estimate of **G**_*t*_ we want E$(G_{t}{|G}_{m}^{*})=G_{m}^{*}$. This can be accomplished by computing:

(7) $$G_{m}^{*}=\beta (G_{m}-I)+I$$

The genomic covariance matrices are diagonally dominant with diagonal elements close to unity. If all diagonal elements in **G**_*m*_is unity the adjustment in (7) corresponds to adjusting estimated variance components by β. In other words the estimate of genetic variance obtained from model (2) is biased downwards with an amount proportional to β.

## Results

### Variance components

A summary of the estimated variance components from all models (1) to (4) are shown in Table 2. Estimates of additive genetic variance using a classical individual animal model (model (1)) ranged from 99.19 to 151.74 on the deregressed scale from the Nordic Genetic Evaluation of Dairy Cattle. The scale of the deregressed proofs are somewhat arbitrary since it depends on the accuracy of breeding values for the bulls included in the cohort used as reference in the standardization predicted breeding values. The ratio of additive genetic variance over the total variance (VR) corresponds to a heritability of the DRP. DRP are basically progeny group means so it would be expected that the proportion of additive genetic variance in DRP increases with increasing progeny group size. In general dairy bulls in the Nordic countries are tested on large groups of progeny. For the production traits, VR were in the range 0.92 for ***milk*** to 0.97 for ***protein***, which clearly indicate that the DRP have high accuracy. For the fitness traits VR were between 0.75 for ***health*** and 0.82 for ***mastitis***. The lower level of VR for the fitness traits is due to the lower underlying heritability of such traits since they are based on the same progeny groups as the production traits.

**Table 2:** Additive genetic and genomic variances for production and fitness traits estimated in models 1, 2, 3 and 4

	**Model 1**		**Model 2**		**Model 3**			**Model 4**
**Trait**	${\hat{\sigma }}_{a}^{2}$	$VR_{a}^{1)}$	${\hat{\sigma }}_{g}^{2}$	$VR_{g}^{1)}$	${\hat{\sigma }}_{g}^{2}$	${\hat{\sigma }}_{a,}^{2}$	$VR_{a+g}^{1)}$	$\sum {\hat{\sigma }}_{a}^{2}$
***Milk***	138.24	0.92	134.18	0.88	119.49	20.87	0.93	115.3
***Fat***	113.10	0.91	109.33	0.87	93.61	22.36	0.94	90.5
***Protein***	143.16	0.97	132.99	0.88	106.67	34.26	0.96	103.0
***Fertility***	151.74	0.78	142.42	0.74	110.38	40.10	0.78	106.5
***Health***	141.57	0.65	136.70	0.63	101.84	42.60	0.66	98.4
***Mastitis***	99.19	0.82	97.30	0.79	81.77	23.67	0.85	79.0

1) Ratio of genetic variance in model over total variance.

For all traits analyzed, the genetic (total genomic) variance estimated in model (2) was lower than the additive genetic variance estimated using the classical individual animal model (1). The reason for this is that the genomic relationship matrix (**G**) do not trace all relationships due to sharing of causative alleles. However, the difference is small and generally the genomic information accounts for between 92% and 98% of the total additive genetic variance depending on the trait in question. These results are well in line with results of [17] and [16] who used regressions on future data to validate their models.

In order to separate effects of polygenic/familial genetic relationships from genomic relationships model (3) were run. In this model the covariance among animals due to additive genetic relationships and due to genomic relationships (markers) both were included. Averaged across all traits total genetic variance estimated in model (3) was 101.7% of total genetic variance estimated in the animal model (1) with a range from 98.4% for ***protein*** to 106.3% for ***mastitis*** (computed from results in Table 2). Model (3) is thus able to explain all genetic variance in the population and estimates of total genetic variance are essentially identical to estimates from the classical individual animal model, which is expected to yield unbiased estimates of additive genetic variance. Averaged over the six traits 77.2% of total genetic variance estimated in model (3) is attributed to genomic relationships among bulls and the remaining 22.8% is attributed to familial genetic relationships among bulls traced via the additive genetic relationship matrix. For the production traits this percentage varied between 75% and 85% and for the fitness related traits it varied between 71% and 78% (percentages computed from Table 2). This indicates that the genomic relationship matrix **G** is able to catch a large proportion of the genetic variation in the population when based on the SNP density used in the current study.

### Additive genetic variance due to individual chromosomes

Results from analysis using model (4), where genomic variance due to individual chromosomes were estimated, is also summarized in Table 2. Only the genomic variance summed over all chromosomes is shown. The chromosomal variance was estimated in 29 individual models where the effect of an individual chromosome and the combined effect of all other chromosomes were estimated. The estimates of variance due to individual chromosomes were then summed over the models. For all traits the sum of variances due to individual chromosomes is slightly smaller than the total genomic variance for each trait in model (3). The sum of chromosomal variance as a percentage of the total genomic variance estimated in model (3) varied between 96% and 97% for all traits (Results not shown). This indicates that covariance between genomic effects on different chromosomes is positive but weak.

**Figure 1:**
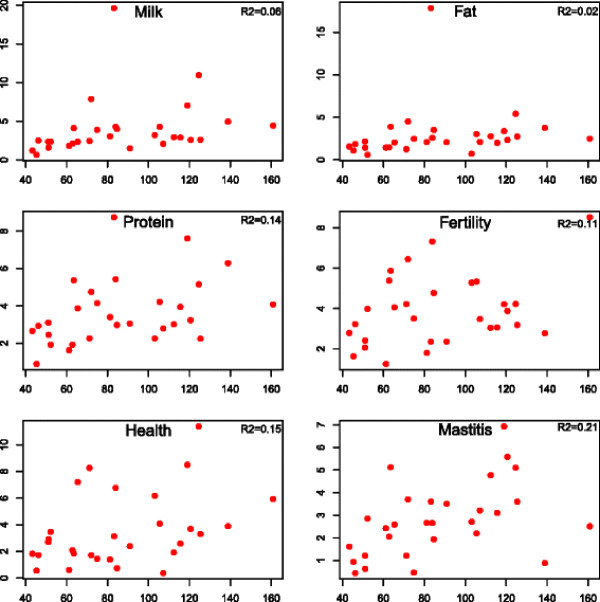
Estimates of genomic variance (y axis) due to individual chromosomes in relation to chromosome length (x axis) in Mb.

Estimates of variance components due to individual chromosomes are shown in Figure 1. The estimates are plotted against the length of individual chromosomes measured as the number of nucleotides (in Mb) between first and last marker on each chromosome. Note that the scale of the estimated variances varies by trait in Figure 1. Very large variances for ***milk*** and ***fat*** were found on chromosome 14 where it is known that a gene of very large effect segregates in the Holstein population [24]. As can clearly be seen from the figure the relation between chromosomal variance and chromosome length is weak. If QTL were evenly distributed over the genome it would be expected that chromosomal variance was closely related to chromosome length. The *R*^2^ values for a linear regression model of chromosomal variance on chromosome length varied between 0.11 and 0.21 (Figure 1). This indicates that QTL are not evenly distributed over the genome. This is clearly the case for ***milk*** and ***fat*** that had very low *R*^2^ due to the major gene segregating as mentioned above. In general the relation between variance due to individual chromosomes might also be dependent on the model and method used as well as the underlying population structure in the data analyzed. 

### Amount of variance explained depending on number of SNPs

**Figure 2:**
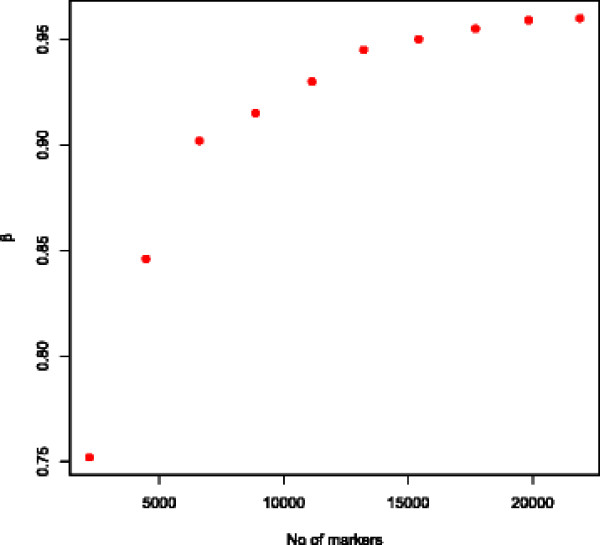
Expected proportion of total additive genetic variance traced by increasing number of markers.

The procedure of [10] were used to estimate the proportion of additive genetic variance that can be explained by markers. This expected proportion (β) is shown in (Figure 2) as a function of number of markers included in the genomic relationship matrix. The expected proportion of additive genetic variance explained by markers is less than 0.85 when the number of markers is below 5 K but increases rapidly until between 15 K and 20 K markers is used in the estimation of genomic variance. Further increases in the number of markers only increase the expected proportion of additive genetic variance explained by markers marginally. The expected proportion of additive genetic variance explained by markers reaches 0.96 when all 44 K markers were used. 

The additive genetic variance explained by different number of markers were investigated using model (2) by varying the number of markers used to compute the genomic relationship matrix. Results averaged over all traits are shown in Table 3. Results using all 44 K markers are the same as in the analysis presented in Table 2 and are repeated here in relative form for reference. As the number of markers included in the analysis decreases the proportion of total additive genetic variance estimated as genomic variance also decreases. As can be seen from Figure 2. The estimates of total genomic variance followed the expectation (proportional to β) quite closely when the numbers of markers included in the genomic relationship matrix were varied.

**Table 3:** **Expected (*β*) and estimated relative amount of additive genetic variance explained by different number of markers**

**No of Markers**	***β***	**Estimated proportion of genetic variance explained by markers**
44012	0.960	0.936
22006	0.930	0.918
11003	0.909	0.880

## Discussion and conclusions

The records analyzed in this paper were **DRP**s which were derived from the routine genetic evaluations of dairy cattle in Denmark, Sweden and Finland. Such **DRP**s are similar to progeny group means adjusted for non-genetic effects. Therefore, a very large proportion of phenotypic variance in analyzed DRP was due to additive genetic effects. For all traits analyzed more than 92% of all additive genetic variance could be explained using 44 K SNP markers. For models including both polygenic additive genetic (pedigree) effects and genomic (marker) effects, the latter accounted for between 71% and 85% of all additive genetic variance. Estimation of genomic variance of each individual chromosome showed that 96%–97% of all genomic (marker) variance could be attributed to individual chromosomes. Inclusion of polygenic familial effects in the models ensured that potential linkage disequilibrium across chromosomes was already taken into account. Most of the additive genetic variance in the population analyzed could be explained by genetic markers. The effect of reducing (increasing) the number of genetic markers on genomic prediction could be predicted by estimating the accuracy of the genomic relationship matrix.

### Variance components

Most of the total phenotypic variance in the traits analyzed was additive genetic due to the use of deregressed proofs, which average out any dominance deviations across multiple daughters. Such proofs, of course, are functions of the procedures and definitions used in the recording system and methods used in the genetic evaluation. For production traits (***milk***, ***fat***, and ***protein***) the additive genetic variance was between 91% and 97% of total phenotypic variance. The high level is expected because of the large sizes of progeny group used in the testing procedures for dairy bulls in all the Nordic countries and because of the high heritability of the underlying traits. For the fitness related traits (***fertility***, ***health***, and ***mastitis***) the relative amount of additive genetic variance is lower reflecting the lower accuracy of fitness related DRP.

The amount of additive genetic variance that can be explained by markers obviously cannot exceed the total additive genetic variance in the population. The classical individual animal model (1) yields unbiased estimates of population additive genetic variance. Comparing results from model (2) (genomic model) with results from model (1) (animal model) clearly illustrates this. For the traits milk and fat 97% of the additive genetic variance can be explained by markers. The estimates of additive genetic variance due to individual chromosomes clearly show that these traits are influenced by a major gene that contributes to the large proportion of additive genetic variance. Statistical models fitting effects of individual marker genes might be a better alternative in these cases.

For ***protein*** and ***fertility*** the amount of additive genetic variance that can be explained by markers is somewhat lower than for milk and fat. This indicate that models used for these traits should include an effect to account for additive genetic effects not accounted for by markers or alternatively more markers are needed to ensure that most of the additive genetic variance can be explained by markers. This is clearly seen from the results of model (3) where both marker effects and classical polygenic familial additive genetic effects were included in the model. However, in general, a large proportion of the total additive genetic variance was explained by markers in this study. This is in considerable contrast to [10] who found that only 45% of additive genetic variance in human height was explained using a marker panel of 294,831 SNPs. One major difference between that study and the current study is that the individuals in the human study were nearly unrelated whereas the bulls in the current study have very many relationships due to the highly structured breeding programs used in commercial dairy cattle. Consequently, the genomic variance ($\sigma_{g}^{2}$) include three different sources of variation: segregation of observed SNPs that cause functional differences in the genes affecting the trait analyzed, co-segregation of markers and causative genes due to physical linkage at population level and linkage disequilibrium at family level. A second difference is that the effective population size in dairy cattle is small with estimates in Danish Holstein of N_e_ = 49 [25], whereas the effective population size in humans is expected to be several orders larger than in dairy cattle.

### Additive genetic variance due to individual chromosomes

When summing over chromosomes the estimates of genomic variance due to individual chromosomes yielded total genomic variances that were similar to the total genomic variance in model (2) where this quantity was estimated directly. The method therefore seems able to yield estimates of genomic variance due to individual chromosomes. Surprisingly the estimates of variance due to individual chromosomes only showed a weak relationship with chromosome length (Figure 1). The results on individual chromosomes are in contrast to results of [26] who found strong relations between chromosomal variance and chromosome length. These authors used a matrix of kinships between individuals as genomic covariance matrix and estimated the variance due to individual chromosomes as the difference between models using all markers and models using all markers but the ones on the specific chromosome in question. Such an indirect procedure is necessary when using kinship matrices, because such matrices normally are singular if they are based on a limited number of markers. We repeated our analysis using methods as in [26]. Generally variance estimates due to genetic similarity matrices are larger than variances due to the genomic relationship matrix used in this paper because of different scales. However, the correlations between estimates of variances due to individual chromosomes and chromosome length were even lower than those presented in this paper and several estimates were negative! This method, therefore, was not further pursued [27]. Also estimate genomic variance due to individual chromosomes. They used a Bayesian approach that allocated equal prior variance to each SNP. Chromosomes with many SNPs, therefore, received more prior variance than chromosomes with few(er) SNPs. They were able to identify genomic regions with larger contribution to genomic variance due to known major genes. However, most SNPs had small effects and therefore there were strong associations between the amount of variance per chromosome and chromosome length or equally number of SNPs per chromosome. Effects of individual SNPs are composed of effects due to co-segregation with closely linked QTL and effects due to LD with QTL elsewhere in the genome, the latter generated by familial relationships in the population. This effect tends to smooth all genomic variance over all SNPs and may therefore give an unclear picture of how much genomic variance can be ascribed to each chromosome. Clearly more research on partitioning of genomic variance into effects of individual chromosomes, chromosome segments or grouping of markers that are expected to be located near causative genes etc. is needed.

### Amount of variance explained by genomic markers

Obviously genetic markers cannot explain more than all the total (additive) genetic variation present in the population. The analysis of the genomic relationship matrices revealed that a large proportion of the total additive genetic variance in the Nordic Holstein population was expected to be explained by a set of 44 K markers. Analysis of both production and fitness related traits showed that the amount of variance accounted for by markers in the Nordic Holstein population was close to the expectations from regression based analysis of the genomic relationship matrix. Estimates of genomic variance closely followed expectation when the number of markers included in computation of genomic relationship matrix was varied. The amount of additive genetic variance that can be explained by genomic markers depends on several factors: Number of markers on causative sites, markers in linkage disequilibrium with causative genes due to close “historical” linkage at population level, and finally linkage disequilibrium among markers and genes at family level, due to the family structure in the population. With 44 K markers spread over the genome the number of markers within causative sites probably is limited. The linkage disequilibrium between markers and causative genes is very dependent on effective population size [28]. The Holstein cattle population has a low effective population size and, therefore, there will be relatively few recombination events in the recent history of the breed. In practice this means that there will be considerable linkage disequilibrium between markers and causative genes. This is also supported by the fact that most of the total additive genetic variation was explained by genomic relationships and not by additive genetic relationships based on pedigree when the model includes both relationship matrices. The family structure in dairy cattle populations creates linkage disequilibrium between markers and causative genes even if they are on different chromosomes and this also helps the markers in being able to explain most of the additive genetic variance in the population.

The analysis of genomic relationship matrices showed that a high proportion of additive genetic variance can be expected to be explained using 44 k genomic markers in this population of dairy cattle. This leaves limited room for further improvements of predictive ability of genomic models by including more markers. One of the current trends in use of genomic markers is to move from 50 K marker chips to 800 K marker chips or even complete sequencing of whole genomes for individual animals. Our results indicate that the advantages of this route may be limited. In fact including several orders of more markers than used in this study may turn out to be counterproductive. Extremely dense markers will include more markers on most causative sites and given knowledge of variation in the causative genes there is no extra information in the remaining markers. Alternative models that better can distinguish between causative genes and non informative markers might be of great value in future

Analysis of the structure of the genomic relationship matrices might be of considerable value in deciding on avenue for future development of typing strategies when using genomic markers. Such analysis also could give extra insight in the effects of population structure and population history on effectiveness of future selection programs using genomic selection in other breeds or in other species.

In summary we estimated the amount of additive genetic variance that can be explained using dense SNP marker panels. In the Holstein population analyzed, almost all the additive genetic variance could be explained using 44 K SNP markers. The amount of additive genetic variance that is expected to be explained by markers could be predicted from analysis of the genomic relationship matrix. Further increases in marker density will have limited effects on predictive accuracy unless better methods distinguishing between markers with real effects and markers with no effect are used. Results presented in this study can be used to determine the weight given to marker relationships and to familial relationships in one step prediction methods where these sources of relationships are combined and in two step methods where information based on genomic relationships must be combined with information form polygenic relationships.

## Abbreviations

GS: Genomic selection; DRP: Deregressed proof; *Milk*: Milk production; *Fat*: Fat production; *Protein*: Protein production; *Fertility*: Female fertility; *Health*: Veterinary treatments for diseases other than mastitis; *Mastitis*: Mastitis.

## Competing interests

The authors declare no competing interests.

## Authors’ contributions

JUJ conceived the study and conducted all analysis, GS developed and implemented algorithms for computing genomic relationship matrices and PM maintained the DMU software package used in the statistical analysis. JUJ edited the manuscript based on extensive input from all authors who have read and approved the final manuscript.
